# Researching complementary and alternative treatments – the gatekeepers are not at home

**DOI:** 10.1186/1471-2288-7-7

**Published:** 2007-02-11

**Authors:** Vinjar Fønnebø, Sameline Grimsgaard, Harald Walach, Cheryl Ritenbaugh, Arne Johan Norheim, Hugh MacPherson, George Lewith, Laila Launsø, Mary Koithan, Torkel Falkenberg, Heather Boon, Mikel Aickin

**Affiliations:** 1 National Research Center in Complementary and Alternative Medicine, Faculty of Medicine, University of Tromsø, N-9037 TROMSØ, Norway; 2 Clinical Research Center, University Hospital of North Norway, N-9038 TROMSØ, Norway; 3 School of Social Sciences & Samueli Institute, University of Northampton, Boughton Green Rd, Northampton NN2 7AL, UK; 4 Department of Family & Community Medicine, University of Arizona, 1450 North Cherry Avenue, Tucson, AZ 85719, USA; 5 Department of Health Sciences, University of York, Heslington, York YO10 5DD, UK; 6 Complementary Medicine Research Unit, Primary Medical Care, Aldermoor Health Centre, Aldermoor Close, Southampton SO16 5ST, UK; 7 Program in Integrative Medicine, University of Arizona, PO Box 245153, Tucson, AZ 85724-5153, USA; 8 Center for Studies of Complementary Medicine, Department of Nursing and the division of International Health (IHCAR), Department of Public Health Sciences, Karolinska Institutet, Alfred Nobels Allé 23, S-141 83 Huddinge, Sweden; 9 Leslie Dan Faculty of Pharmacy, University of Toronto, 19 Russell Street Toronto, Ontario, M5S 2S2, Canada

## Abstract

**Background:**

To explore the strengths and weaknesses of conventional biomedical research strategies and methods as applied to complementary and alternative medicine (CAM), and to suggest a new research framework for assessing these treatment modalities.

**Discussion:**

There appears to be a gap between published studies showing little or no efficacy of CAM, and reports of substantial clinical benefit from patients and CAM practitioners. This "gap" might be partially due to the current focus on placebo-controlled randomized trials, which are appropriately designed to answer questions about the efficacy and safety of pharmaceutical agents. In an attempt to fit this assessment strategy, complex CAM treatment approaches have been dissected into standardized and often simplified treatment methods, and outcomes have been limited.

Unlike conventional medicine, CAM has no regulatory or financial gatekeeper controlling their therapeutic "agents" before they are marketed. Treatments may thus be in widespread use before researchers know of their existence. In addition, the treatments are often provided as an integrated 'whole system' of care, without careful consideration of the safety issue.

We propose a five-phase strategy for assessing CAM built on the acknowledgement of the inherent, unique aspects of CAM treatments and their regulatory status in most Western countries. These phases comprise:

1. Context, paradigms, philosophical understanding and utilization

2. Safety status

3. Comparative effectiveness.

4. Component efficacy

5. Biological mechanisms.

**Summary:**

Using the proposed strategy will generate evidence relevant to clinical practice, while acknowledging the absence of regulatory and financial gatekeepers for CAM. It will also emphasize the important but subtle differences between CAM and conventional medical practice.

## Background

The use of complementary and alternative medicine (CAM) has increased considerably in Western industrialized nations over the last 25 years. In the USA, the expenditure is approximately $30 billion per annum, surpassing current out-of-pocket expenditures for conventional treatments by primary care physicians [1,2]. CAM treatment modalities include a variety of approaches (i.e., acupuncture, homeopathy, herbal medicine, massage, reflexology, Reiki healing etc.), many of them based on theories that differ markedly from conventional Western biomedicine. The assessment and management of illness often includes a more detailed interest in patients' wellbeing, as well as recommendations concerning lifestyle and their quality of life as a whole [3].

Patients are selective in their choice of CAM treatments [4], usually abstaining from exclusive use of CAM in acute life-threatening conditions. Rather, CAM is mainly used in addition to conventional care for chronic and some acute health conditions, for disease prevention and for maintaining wellness. For example, more than half of all breast cancer patients use some form of CAM complementary to conventional medicine, most often with a palliative intent [5,6].

Many physicians do not understand why large segments of their patients use CAM when research generally has failed to provide decisive evidence of efficacy. Patients seem to make these treatment choices based on the qualities of the provider, desire for "individualized" treatments, and their perception of overall effectiveness rather than efficacy [7-11].

There is apparently a "gap" between the results of randomized controlled trials (RCT) showing little or no effect and the widespread use and reports of beneficial outcomes of CAM treatment [12,13]. If we were to assume that patients are not completely misguided, then we would need to look closely at the research strategies utilized in the CAM field and try to understand the reasons for the gap.

The purpose of this paper is therefore to

1. Explore the strengths and weaknesses of conventional biomedical research strategies and methods as applied to CAM

2. Suggest a new research framework for assessing these treatment modalities.

## Discussion

### Gatekeeping and regulating the use of CAM interventions

A majority of CAM research to date has used the research strategy employed and developed by clinical pharmacologists to document, in a prescribed sequential pattern, the quality, dose, safety, efficacy, and eventual effectiveness of a drug prior to its general release. In this model, governmental regulatory offices (e.g., USA: FDA, EU: EMEA) act as gatekeepers. Health insurers, including governmental single-payer funders, only reimburse for drugs meeting the appropriate criteria. An important principle in this research-regulatory-utilization model is that research determines which drugs are approved for generalized clinical use and are paid for by health insurers. There is also a growing network of international Health Technology Assessment Boards that seek consensus about the use of medical procedures, and some suggest this approach should be extended to the area of diagnostic tests [14].

Previously, CAM researchers have largely assumed that these same pharmacological research methods and regulatory-reimbursement models can be followed in the evaluation of CAM. Much to the frustration of many clinical researchers, the availability of CAM treatments which are affordable with little or no reimbursement, however, does not seem to be amenable to the same rules as those we seek to apply to conventional medicine. Even if studies show that a CAM treatment has no effect, it does not necessarily disappear from the marketplace. We suggest that this is because the CAM market has had no statutory body (gatekeeper) that ensured the quality, safety, efficacy and effectiveness of CAM treatments before they appear on the "market". Further, since few health insurers reimburse for CAM treatments, there are few financial gatekeepers. Millions of patients in developed countries have experienced the effect of CAM treatments provided in contrast to, or more likely in addition to, the care they have received in conventional medicine [15-19]. The situation is thereby characterized by widespread patient experience of treatment outcomes combined with little research and no regulatory or financial gate-keeping activity.

### Why is a different research strategy needed?

In conventional medicine we rarely question the efficacy or effectiveness of the overall therapeutic approach to the treatment of sick people. We take for granted that seeking advice from a health care professional is a good way of dealing with diverse health problems. Research in conventional medicine therefore focuses on choosing the best tools for health professionals to use. These tools include drugs, diagnostic methods, and surgical procedures, and employ a methodology often resulting in a one-size-fits-all therapeutic prescription. Conventional research may, however, have overlooked that the clinical effect of most therapies are overestimated when studied under optimal circumstances on susceptible, cooperative patients. Despite this, the randomized controlled trial is an important method when making decisions about tools to include in the toolbox of conventional medicine.

Because "conventional" researchers have done most of the CAM research, it is not surprising that CAM research has traveled down the same path as conventional medicine. It has tested the specific efficacy of what conventional researchers believe to be the active components of a therapy, often discounting synergistic effects. In addition to providing a predominantly individualistic treatment approach, many CAM therapists hold that CAM treatments cannot be split up into parts that can be investigated separately. They argue that the total effect adds up to more than the sum of its parts.

If CAM is to be evaluated comprehensively, one needs to extend the research focus to all aspects of the treatment approach [20]. To study only the specific effect of needling in acupuncture isolated from other interventions initiated by the acupuncturist, or the effect of a single, isolated homeopathic remedy separated from other aspects of homeopathic practice, is to neglect other potentially important components of these interventions [21-25]. Furthermore, clinical research teams should only venture into this area with a thorough contextual and philosophical understanding of the CAM treatment paradigm and its clinical use. Plunging into studies of efficacy that involve isolated detailed components of a treatment approach without thoroughly understanding its context is destined to failure and irrelevance no matter what the results show. These issues point to the need for a different, and more complex, research strategy for the CAM field.

### A suggested research strategy

**Figure 1:**
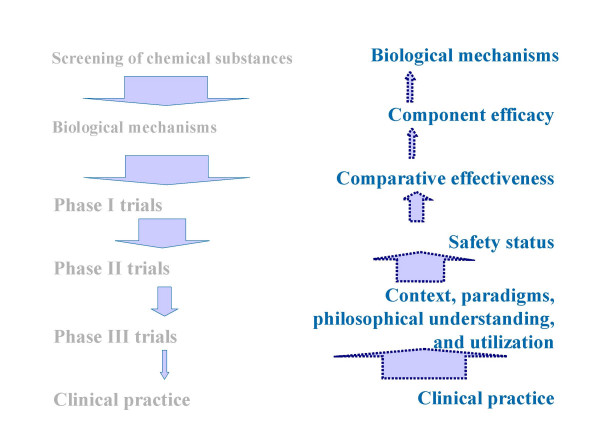
**Research strategies in drug trials and CAM (proposed)**. Phases that contrast the proposed phased research strategy in CAM (dark arrows) with that conventionally used in drug trials (light arrows).

The most important unique characteristics of CAM are the absence of gatekeepers and the complexity of individually tailored treatments. Our proposed strategy does not contain new methodological elements, but organizes existing elements in a way that is tailored to pragmatic clinical practice (Figure 1). The strategy is not meant as a strictly chronological sequence defining when specific research phases should occur, but as a framework to guide CAM research, illustrating the necessary building blocks required for a rigorous evidence base.

Although we have developed this strategy within a CAM context, it is appropriate for any treatment approach that has developed in and from clinical practice, and whose specific treatment tools are unregulated. In conventional medicine this might include nursing, health psychology, counseling and some aspects of general practice. These areas struggle, as does CAM, to establish a rigorous research framework around which their research efforts can be organized [26-28].

### Phase1: Context, paradigms, philosophical understanding and utilization (What is going on?)

The cornerstone of the suggested research strategy is to understand the process and assumptions within a particular therapy, often using an inductive research approach [29-34]. Researchers need to understand what the treatment procedure is, how many variations there are, what philosophical foundations underlie it, its ideas about health and disease, its contextual framework and key treatment components. They also need to know how many and what segments of the patient population use it, and for what conditions. Many research questions can be asked within phase I, including perceived benefit of the treatment, cost of treatment, qualifications of the providers etc.

### Phase 2: Safety status (Is it safe?)

Safety issues are important in the treatment of any illness, but in the area of CAM they cannot be emphasized strongly enough. The inherent risks of the diseases or illnesses for which patients seek CAM are generally low. The treatments given should therefore carry a low risk of adverse effects. CAM treatments have often been claimed to be without risk, but adverse effects in CAM are more than occasional case reports. [35]. The methods of choice to study safety would be similar to the detection of adverse events associated with pharmaceutical treatments. The field of acupuncture has provided a thorough and rigorous risk assessment [36-39] based on these principles and the other fields within CAM need to follow suit [40-42].

### Phase 3: Comparative effectiveness (What is the system effectiveness?)

Patients are seeking CAM as a treatment system. Research needs to examine the outcomes of these treatments both in combination with, and as alternative, to conventional care. Thus randomized, controlled pragmatic trials are needed [43,44], wherein the specifics of the system are not disassembled, but the system under study is allowed to function as it is clinically practiced, including the urgently needed evaluation of cost-effectiveness [45]. The pragmatic trial design has been used in the study of acupuncture treatment of chronic pain [46], and the methodology is also widely used in conventional medicine, for example in dietary intervention studies such as the Women's Health Initiative [47]. In these trials, patients are randomly assigned to treatment alternatives which may include alternative viable whole systems, conventional treatment or no treatment. The trials may also evaluate whole system interventions that are implemented under protocols that specify individualized treatments based on specific patient characteristics, following the patterns of care found in the community. While blinding of the treatment providers and patients/subjects with regard to treatment allocation is usually not feasible, blinding of the outcomes evaluators can ensure an unbiased comparison of the outcome assessment.

In conventional medicine, a treatment's efficacy is often determined before assessment of its effectiveness. It is considered unethical to include non-efficacious treatments in the real world treatment of patients. Patients are, however, already using CAM treatments and thus effectiveness studies can be used to guide decisions about the necessity of studying the efficacy of specific components. Researchers have voiced this view in several countries over the last few years [20,48,49]. It was even emphasized in the much-criticized recent meta-analysis of homeopathy [50]: "Clearly, rather than doing further placebo-controlled trials of homoeopathy, future research efforts should focus on the nature of context effects and on the place of homoeopathy in health-care systems".

Included in this phase, and bordering on the next phase would be studies to evaluate how limited a whole package of care can be while still retaining its overall effectiveness ("Occam's razor"). There are many components within a CAM treatment approach, but are there any that we can eliminate and at the same time retain or improve overall treatment effectiveness?

### Phase 4: Component efficacy (What is the efficacy of a specific component of the therapy?)

This is the area that has received most attention and research money to date, and while it is important, it is not the starting point in our model. The methods of choice are often double-blind randomized controlled trials. The well-established documentation of acupuncture/acupressure stimulation of one acupuncture point in the treatment of chemotherapy-induced nausea/vomiting is such an example [51]. It is, however, important to recognize that results from such research cannot be used to document or disprove the effectiveness of a "whole system" treatment.

### Phase 5: Biological mechanisms (How can treatment outcomes be explained biologically?)

We want, and need, to understand the pathways and mechanisms through which treatments exercise their influence [52]. This has been and is being explored for acupuncture as an anti-emetic [53]. We must however realize that treatment outcomes, both at the system and component level, can be documented before the biologic mechanisms are understood, as has been the case for several non-surgical conventional therapies. The most prominent example from conventional medicine is probably aspirin. The anti-inflammatory and pain-killing properties of aspirin were discovered long before it became known that aspirin influences prostaglandin synthesis.

## Summary

CAM is not simply a new array of therapeutic tools that need to be evaluated; it presents other ways to think about disease and therapeutics, and consequently new ideas about how research should be strategically developed. In this article we have suggested two ways of taking this forward. First, the absence of statutory and financial gatekeepers for CAM presents several issues that need to be considered closely. Secondly, the structure of CAM research should be different, in subtle but important ways. We have provided some suggestions as to how this alternative research strategy could be structured keeping in mind that the ultimate goal for all approaches to treatment is to provide effective medical interventions at reasonable cost and without harm.

## Competing interests

The author(s) declare that they have no competing interests.

## Authors' contributions

VF and SG conceived of the study. VF drafted the manuscript. All authors have contributed to the paper by offering textual elements and decisive references, contributing to the critical discussion that fostered the article, and a critical reading. All authors read and approved the final manuscript.

## Pre-publication history

The pre-publication history for this paper can be accessed here:


